# Bayesian network analysis revealed the connectivity difference of the default mode network from the resting-state to task-state

**DOI:** 10.3389/fncom.2014.00118

**Published:** 2014-09-24

**Authors:** Xia Wu, Xinyu Yu, Li Yao, Rui Li

**Affiliations:** ^1^College of Information Science and Technology, Beijing Normal UniversityBeijing, China; ^2^State Key Laboratories of Transducer Technology, Shanghai Institute of Technical Physics, Chinese Academy of SciencesShanghai, China; ^3^State Key Laboratory of Cognitive Neuroscience and Learning, Beijing Normal UniversityBeijing, China; ^4^Center on Aging Psychology, Key Laboratory of Mental Health, Institute of Psychology, Chinese Academy of SciencesBeijing, China

**Keywords:** default mode network (DMN), effective connectivity (EC), functional connectivity (FC), Bayesian network (BN), functional magnetic resonance imaging (fMRI)

## Abstract

Functional magnetic resonance imaging (fMRI) studies have converged to reveal the default mode network (DMN), a constellation of regions that display co-activation during resting-state but co-deactivation during attention-demanding tasks in the brain. Here, we employed a Bayesian network (BN) analysis method to construct a directed effective connectivity model of the DMN and compared the organizational architecture and interregional directed connections under both resting-state and task-state. The analysis results indicated that the DMN was consistently organized into two closely interacting subsystems in both resting-state and task-state. The directed connections between DMN regions, however, changed significantly from the resting-state to task-state condition. The results suggest that the DMN intrinsically maintains a relatively stable structure whether at rest or performing tasks but has different information processing mechanisms under varied states.

## Introduction

The brain's default mode network (DMN), characterized by increased neural activity in specific brain regions during resting-state compared with goal-oriented tasks (Raichle et al., 2001; Greicius et al., 2003; Raichle and Snyder, 2007), has generated a significant amount of interest in recent brain imaging studies. There is a growing recognition that the DMN plays a significant role in the brain, including such functions as self-referencing, autobiographical memory retrieval, consolidation of past experience and future preparation (Gusnard and Raichle, 2001; Svoboda et al., 2006; Hahn et al., 2007; Buckner et al., 2008). A number of studies have been conducted attempting to elucidate the DMN's anatomy (Mazoyer et al., 2001; Greicius et al., 2003; Fox et al., 2005; Fransson, 2005), working mechanism, its interaction with other neural systems (Mazoyer et al., 2001; Greicius et al., 2004; Rombouts et al., 2005; Wang et al., 2006; Margulies et al., 2007; Buckner et al., 2009; Demirci et al., 2009; Li et al., 2009, 2012; Stevens et al., 2009; Liao et al., 2010; Wu et al., 2011) and its relevance to disease (Greicius et al., 2004; Rombouts et al., 2005; Wang et al., 2006).

Recently, an increasing amount of imaging computing methods have been introduced to explore the connectivity architecture of the DMN. Functional investigation of the DMN based on the inter-regional correlation and independent component analysis (ICA) lead to the consensus that the DMN is composed of specific brain regions, including the posterior cingulated cortex (PCC), medial prefrontal cortex (MPFC), inferior parietal cortex (IPC), inferior temporal cortex (ITC), and hippocampus (HC). Among these regions, the PCC, MPFC and the bilateral IPC were demonstrated to show the strongest regional correlations with each other and the highest metabolism in the network (Raichle et al., 2001; Greicius et al., 2003; Buckner et al., 2008). Moreover, these regions also showed nearly consistent complete convergence across multiple approaches which define the anatomical organization of the DMN including task-based deactivation studies and resting-based spontaneous activity studies using PET and fMRI techniques (Raichle et al., 2001; Greicius et al., 2003; Fransson, 2005; Raichle and Snyder, 2007; Buckner et al., 2008). These regions have therefore been suggested as the hubs of the network (Buckner et al., 2008). In addition, the PCC, MPFC and IPC regions from the DMN were also demonstrated as the important hubs in the whole cerebral cortex (Buckner et al., 2009). These hubs were supposed to function to coordinate and integrate the information processing of spatially distributed regions or distinct neural systems in the brain (Buckner et al., 2008, 2009; Li et al., 2009, 2012).

In addition to studying the undirected functional connectivity relationship between brain regions within the DMN, a variety of directed effective connectivity analysis approaches including the Granger causality mapping (GCM) (Goebel et al., 2003) and Bayesian network (BN) (Zheng and Rajapakse, 2006; Wu et al., 2011), have been introduced in an attempt to explore the information exchange mechanism of the DMN. GCM uses a vector autoregressive model to analyze the functional interactions among regions and is a pair-wise connectivity analysis rather than a global representation of a neural system. Jiao et al. (2011) investigated the Granger causal relations in the DMN and found a highly consistent hierarchical distribution of the activity in the network, with the highest level in the PCC and MPFC and with the lowest level in the ITC. The BN learning approach, which is a popular technique that has been widely applied to complex systems for uncertainty reasoning and data analysis, is capable of learning the global effective connectivity pattern rather than the pair-wise connectivity, without any prior assumptions (Zheng and Rajapakse, 2006). Ever since Zheng et al. (Zheng and Rajapakse, 2006) firstly applied the BN learning approach to characterize the effective connectivity patterns among brain regions in fMRI in the process of investigating silent word reading and counting Stroop tasks, the BN approach has been widely applied to fMRI data as a tool to determine the conditional dependencies between brain regions (Zheng and Rajapakse, 2006; Rajapakse and Zhou, 2007; Kim et al., 2008; Li et al., 2008, 2009). In our previous study of the DMN using the BN method (Li et al., 2009, 2012, 2013), we also demonstrated distinct connectivity patterns between the PCC, MPFC, IPC and additional regions including ITC and HC. The activity in the PCC, MPFC and IPC were found to depend more on the network and show a higher degree of local interactivity relative to other DMN regions (Li et al., 2013). In combination with the functional connectivity of the DMN, the directed connectivity of the network has further suggested that the functional architecture of the network is hierarchically organized into at least two interacting subsystems, with the PCC, IPC, and MPFC representing anatomic and functional keys or hubs in the DMN and the ITC together with the HC representing the non-hubs (Buckner et al., 2008; Li et al., 2012, 2013).

The investigations of the DMN's connectivity to date are of great significance to understanding the working mechanism of DMN, but overall, the current understanding and awareness of the DMN have mainly been obtained by investigating the temporal correlations between regions in the resting-state network (Friston, 1994) or the relationship between the DMN and neuropsychiatric disorders such as Alzheimer's disease and depression (Greicius et al., 2004; Rombouts et al., 2005; Wang et al., 2006). In comparison, fewer studies have addressed the directed connectivity architecture of the network and compared the network between resting-state and other different task-state conditions. It is suggested that probing deeply into the DMN and detecting the differences between the resting-state and the task-state could help us better comprehend the working mechanisms implied in this network. Adhering to this goal, some studies have started to focus on the relationship between the task activation and resting-state activities (Smith et al., 2009; Ma et al., 2012). They have found that the full repertoire of functional networks utilized by the brain in action is continuously and dynamically “active” even when at “rest.” However, we still understand very little about the connection framework of the DMN and its information exchange during different working states. Thus, it is essential for us to further study the difference of the network between the resting-state and task-state conditions.

Specific to this study, given the accumulating number of studies investigating the DMN, we sought to employ the BN learning approach (Friedman et al., 1997; Heckerman, 1998; Zheng and Rajapakse, 2006; Rajapakse and Zhou, 2007; Kim et al., 2008; Li et al., 2008, 2009, 2012, 2013) to construct an effective connectivity model of the DMN in 14 healthy subjects during both resting-state and task-state, and then, we compared the organization pattern and connection characteristics of the brain regions within the DMN between these two states. In addition to the BN effective connectivity analysis of the DMN, we further introduced a random permutation test to examine the connectivity difference between the resting-state and the task-state conditions.

## Materials and methods

### Subjects and task

Fourteen healthy volunteers [8 males and 6 females, ages between 19 and 26 years (Mean ± SD: 21.1 ± 3.74 years old), right-handed] participated. Handedness was determined by the Edinburgh Inventory. All participants were native Chinese (Mandarin) speakers with no history of psychiatric or neurological abnormalities. All participants had normal or corrected to normal vision through the use of MRI-compatible lenses. The purpose of the study was explained to the participants, and each of them provided written informed consent approved by the Research Ethics Committee of the State Key Laboratory of Cognitive Neuroscience and Learning, Beijing Normal University (BNU), prior to the experiment.

All of the subjects were first instructed simply to keep their eyes closed and not to think of anything in particular for the resting-state scan. Then, a semantic judgment task, written word semantic judgment (WJ), was followed to collect the data during task condition. The participants were asked to judge whether the visually presented items (e.g., gun, sheep) were semantically dangerous or not. A positive response was indicated via key pressing by the subject's right hand, whereas a negative response was indicated by his/her left hand. The participants were asked to respond as quickly and accurately as possible. This task is described in detail in our previous publication (Wu et al., 2009).

### Data acquisition

MRI scanning was performed on a 1.5-Tesla Siemens whole-body MRI system at Xuan Wu Hospital in Beijing. Gradient echo-planar imaging was used to acquire 20 axial slices (6 mm thickness, 1.8 mm gap, field of view, 220 × 220 mm^2^; matrix size, 64 × 64; repeat time, 2000 ms; echo time, 50 ms; flip angle, 90°; 132 repetitions per time series). A high-resolution T1-weighted 3D MRI sequence with the following parameters was used: 1.9 mm thickness; 0.95 mm gap; repeat time, 1970 ms; echo time, 3.93 ms; and flip angle, 15°.

### Data preprocessing

For each participant, the original first five-time functional images were discarded to allow for equilibration of the magnetic field. All of the preprocessing steps were performed using the Statistical Parametric Mapping program (SPM8; http://www.fil.ion.ucl.ac.uk/spm/). They included within-subject inter-scan realignment, between-subject spatial normalization to a standard brain template in the Montreal Neurological Institute (MNI) coordinate space and smoothing by a Gaussian filter with a full width at a half maximum of 8 mm. Following this, detrending and temporal band-pass filtering of the fMRI data were performed in order to reduce the effects of low-frequency noise (Biswal et al., 1995). To exclude the impact of head movement and several potential nuisance signals on the regional connectivity (Friedman et al., 2000; Power et al., 2012; Van Dijk et al., 2012), we then backed off the possible sources of artifacts of the fMRI data, including six head-motion profiles, global signal, white matter signal, and cerebrospinal fluid (CSF) signal via the Resting-State fMRI Data Analysis Toolkit (REST; http://restfmri.net).

### Group independent component analysis

Group ICA is widely used to separate patterns of task-activated neural networks, image artifacts and physiologically generated independent components (ICs) in a data-driven manner. The preprocessed resting-state and task-state data of all participants were separately entered into the Group ICA program in the fMRI Toolbox (GIFT, http://icatb.sourceforge.net/) for the separation of DMN and the determination of DMN regions for BN analysis. The Group ICA program included twice principal component analysis (PCA) for reduction of the fMRI data dimensions, ICA separation and back-reconstruction of the ICs and the corresponding mean time course for each subject (Calhoun et al., 2001). The optimal number of principal components, 15 for resting-state data and 16 for task-state data, were estimated based on the minimum description length (MDL). In the first round of PCA, the data for each individual subject were dimension-reduced to the optimal number temporally. After concatenation across subjects within groups, the dimensions were again reduced to the optimal numbers via the second round of PCA. Then, the data were separated by ICA using the Extended Infomax algorithm (Lee et al., 1999). After ICA separation, the mean ICs and the corresponding mean time courses over all of the subjects were used for the back-reconstruction of the ICs and time courses for each individual subject (Calhoun et al., 2001).

Finally, the independent component that best matched the DMN was selected for both the resting-state and task-state data. Following this, one sample *t*-test (*p* < 0.001, corrected by false discovery rate, FDR) was then performed to separately determine the DMN for the resting-state and task-state data. Between-group DMN differences were determined by two-sample *t*-test (FDR corrected, *p* < 0.005).

### Bayesian network analysis

To determine the regions for the subsequent Bayesian network analysis, we identified eight DMN regions as regions of interest (ROIs) for both the resting-state and task-state data separately. Each ROI was defined as a 6-mm sphere centered on the local maximum functional connectivity (FC) cluster in the DMN map from the ICA analysis. We overlaid the results of one sample *t-test* onto these eight spheres to obtain the final ROIs. The list of spheres' center coordinates are shown in Table 1. The ROIs were then entered into the BN analysis for the construction of EC patterns of DMN. The averaged time series over the voxels in each ROI of every subject were extracted and then linked individual-by-individual to represent the time series of each ROI for the BN analysis.

**Table 1:** **The ROIs defined for BN analysis**.

**Condition**	**Brain region**	**MNI coordinate**			**BA**	***T*-value**
		***x***	***y***	***z***		
Resting-state	PCC	−6	−60	28	31	18.17
	Left IPC (lIPC)	−42	−66	44	40	15.45
	MPFC	1	50	24	9, 10	10.97
	Right IPC (rIPC)	48	−57	36	39, 40	9.25
	Right ITC (rITC)	63	−27	−20	20	7.43
	Left ITC (lITC)	−59	−21	−20	20, 21	6.43
	Left HC (lHC)	−24	−12	−28	35	5.82
	Right HC (rHC)	24	−30	−16	36	6.77
Task-state	PCC	−1	−63	24	7, 31	15.81
	Left IPC (lIPC)	−45	−69	39	39, 40	9.38
	MPFC	−6	54	28	9	15.18
	Right IPC (rIPC)	52	−66	28	39	5.12
	Right ITC (rITC)	54	6	−36	21	9.43
	Left ITC (lITC)	−51	−9	−28	20, 21	10.39
	Left HC (lHC)^*^	−24	−12	−28	35	5.82
	Right HC (rHC)^*^	24	−30	−16	36	6.77

* The lHC and rHC are not shown to be active in the task-state data at p < 0.001 with FDR corrected. To ensure the consistency of the network structure, we choose the resting-state data's 6-mm sphere center coordinates of the two regions for task-state data.

A BN model is a directed acyclic graph (DAG) that encodes a joint probability distribution over a set of random variables *X* = {*X*_1_, *X*_2_, …, *X_n_*}, in which nodes represent the brain regions for the connectivity analysis, and arcs denote the conditional dependence relationships between these regions. The dependencies are qualified by the conditional probability of each region node given its parent region nodes in the network. In addition, the absence of arcs represents conditional independencies among these regions. The BN graph encodes the Markov assumption. That is, each node is independent of its non-descendants, given its parent nodes in the network (Friedman et al., 2000). The BN can learn the global connectivity patterns for complex systems without any prior knowledge in a data-driven manner.

In our study, nodes in the BN represent ROIs from the DMN, the time series from which is assumed to follow a linear Gaussian conditional distribution. The nodes *X_i_* (*i* = 1, 2, …, *n*) represents the *ith* ROI (*n* = 8), and the conditional probability density of *X_i_* given its parents *Pa* (*X_i_*) can be given by

$$P\left(X_{i}|Pa(X_{i})\right)=\frac{1}{\sqrt{2\pi }\sigma_{i}}exp\left[-\frac{1}{2\sigma_{i}^{2}}{\left(x_{i}-u_{i}\right)}^{2}\right]$$

Where *u_i_* and σ_*i*_ are, respectively, the conditional mean and conditional variance of child node *X_i_* given its parent nodes, and $u_{i}=\mu_{i}+\sum_{X_{p}\in Pa(X_{i})}b_{p}(x_{p}-\mu_{p}).\;b_{p}$ is the connection weight coefficient from parent node *X_p_* to *X_i_* that quantifies the strength of relationship between them (Geiger and Heckerman, 1994); μ*_i_* is the unconditional mean of node *X_i_*; μ*_p_* is the unconditional mean of parent node *X_p_*. The joint probability distribution of *X* = {*X*_1_, *X*_2_, …, *X_n_*} is defined as a multivariate Gaussian as listed below:

$$P\left(X_{1},X_{2},\cdots ,X_{n}\right)=\prod_{i=1}^{n}P\left(X_{i}|Pa(X_{i})\right)$$

In other words, a determined linear Gaussian BN is the same as a set of multivariate linear regression equations and each node *X_i_* can be considered as the linear regression of its parent nodes *Pa* (*X_i_*) (Shachter and Kenley, 1989).

The BN is typically viewed as a model selection problem (Zucchini, 2000), which aims to find the network that best characterizes the conditional dependencies represented by the observation data. In general, there are two different model selection approaches: constraint-based approach and score-based approach (Spirtes et al., 2000). The constraint-based approach performs a number of hypothesis tests on the independent relations between variables firstly, and next searches for the network structure which is best consistent with the relations between brain regions observed from data. The score-based approach makes use of scoring metrics to guide the search process and choose the network structure which maximizes the scoring function as the optimal selection. Currently quite popular scoring functions include the MDL, Bayesian information criterion (BIC) and Akaike information criterion (AIC) (Akaike, 1974; Rissanen, 1978; Schwarz, 1978).

To learn the EC among the DMN regions, we employed the BIC-based learning approach. The BN model that maximized the BIC score among the space of possible candidates was selected as the best fit network. The BIC is given by formula

$$BIC\left(G\text{ | }D\right)\approx \log P\left(D\text{ | }G,\Theta^{*}\right)-\frac{d}{2}\log m$$

in which the first term log *P* (*D* | *G*, Θ^*^) is the maximized log-likelihood of data *D* conditional on Θ^*^, which measures the degree of goodness for a given Θ^*^, the maximum likelihood (ML) estimation of parameters. The second term $\frac{d}{2}\log m$ is a penalty on the learned network complexity. Parameter *d* is the number of independent parameters, and *m* is the number of the data samples.

Then, we used the L1-Regularization Paths algorithm (Schmidt et al., 2007) and the maximum likelihood (ML) estimate implemented in the collections of Matlab functions written by Murphy et al. (http://www.cs.ubc.ca/~murphyk/Software) to learn the DAG structure and parameters of the BN model, respectively, for the resting-state and task-state. A step-wise regression procedure was then performed to test the significance of connections in the learned BN model of DMN. This significance test approach was based on the fact that the identified Gaussian BN was equivalent to a set of multivariate linear regression equations. That is, each node in the BN model can be considered as a linear regression of its parent nodes with connection weights as the regression coefficients (Shachter and Kenley, 1989; Li et al., 2009). Thus, the statistical significance of the regression coefficients can be tested (*p* < 0.05). Finally the set of regression equations with significant weights were in turn expressed in the form of BN graph (Li et al., 2009, 2013; Wu et al., 2011), which was the determined as the effective connectivity model of DMN.

### Effective connectivity comparison between the resting-state and task-state

For the constructed BN model of DMN, it was also our interest to examine the difference of the effective connectivity between the resting-state and the task-state groups via a randomized permutation. The null hypothesis is that there is no significant difference of the BN connectivity weight coefficients between the resting-state and the task-state groups. We take the differences of the connection weight coefficients between the two conditions as the statistical measure. The reference distribution is obtained by calculating all possible values of the test statistic under rearrangements of the group labels on the observed fMRI datasets. The statistics for the real two group samples were calculated first. Then at each iteration of the test process, the subject-group membership was randomly assigned for each subject. A BN model for each rearranged group was constructed, and the differences of the connection weight coefficients between the two rearranged groups were calculated. We ran a total of 1000 permutations and assessed the sample distributions for these statistics. Finally, the probabilities of the connections in the BN model of resting-state group that were stronger than the ones in the task-state group as well as the probabilities of the connections in the model of task-state group that were stronger than the ones in the resting-state group were examined for each of the connections presented in the BN model for the resting-state group or task-state group.

## Results

### Functional connectivity result of the DMN

**Figure 1:**
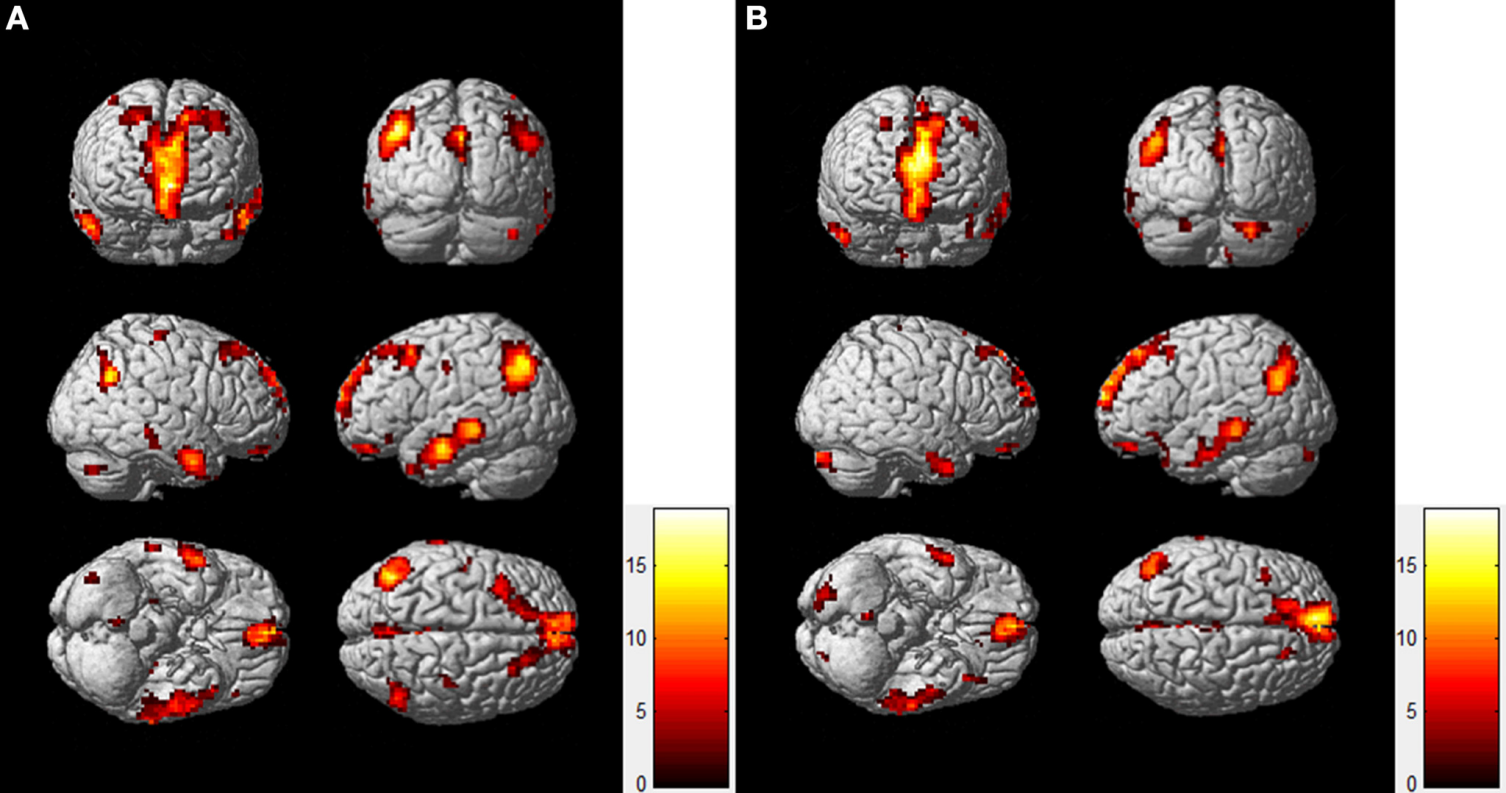
**DMN Maps of the resting-state (A) and task-state (B)**.

Figure 1 shows the group DMN results in the resting-state (A) and task-state (B), respectively, detected by Group ICA followed by one-sample *t*-test with a *p* < 0.001 (FDR corrected). The DMN in both the resting-state and task-state includes the PCC, MPFC, bilateral IPC, ITC, and HC. To determine the regions for subsequent EC analysis of the DMN in both the resting-state and task-state groups, we defined the eight brain regions mentioned above as ROIs in these two groups (Table 1).

### Between-group DMN functional connectivity difference

**Figure 2:**
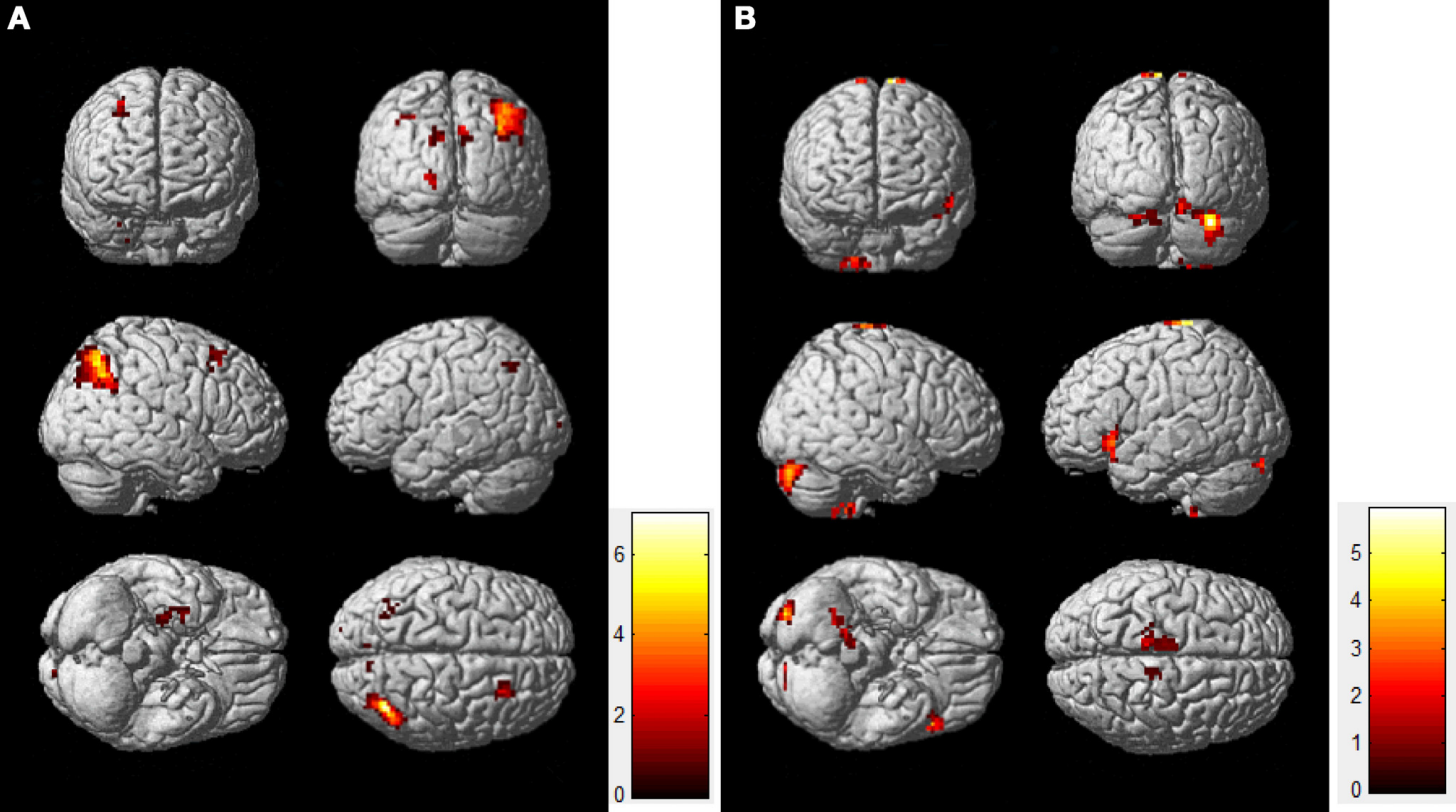
**Functional connectivity difference of the DMN between resting-state and task-state**. The left panel **(A)** represents regions that show increased connectivity during resting-state (“rest>task”), and the right panel **(B)** represents the opposite case (“task>rest”).

To compare the functional connectivity difference of the DMN between the resting-state and task-state conditions, we performed a two-sample *t*-test (FDR, *p* < 0.005) on individual DMN maps between the two groups. Figure 2 displays the functional connectivity differences of the DMN between the two states. The regions including the lIPC, rIPC, and PCC display increased functional connectivity in the resting-state compared with the task-state (“rest>task”), whereas the lITC displays increased functional connectivity during the task-state compared with the resting-state (“task>rest”).

### BN-based effective connectivity of the DMN

**Figure 3:**
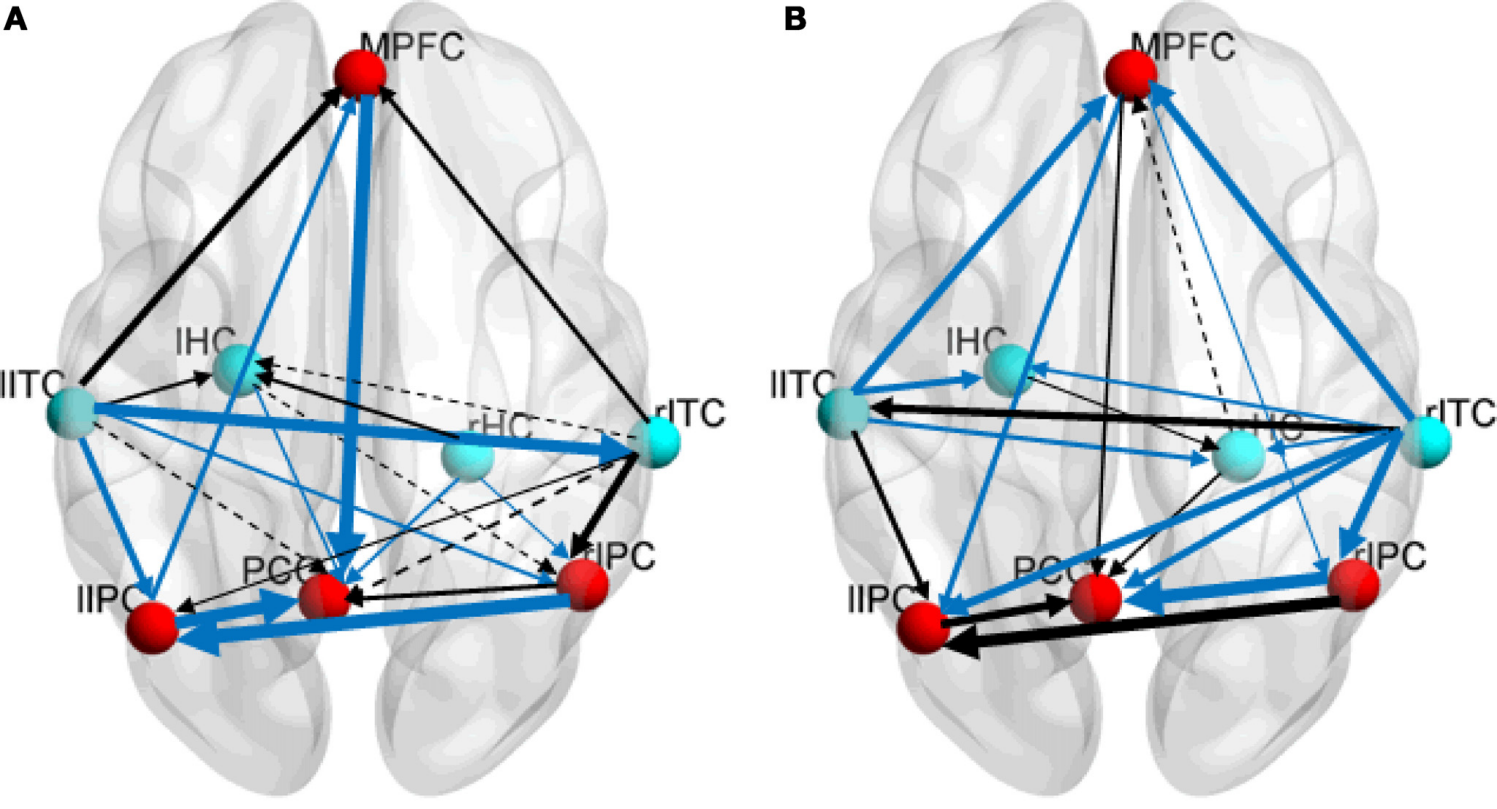
**Effective connectivity models of DMN during the resting-state (A) and the task-state (B)**. Nodes responsible for the hubs and the non-hubs are shown in red and blue, respectively. Solid and dashed arcs are, respectively, for positive and negative connections. The line width is proportional to the connection weights. The connections in the resting-state group stronger than the ones in the task-state group (“rest>task”) are shown in blue in the left panel **(A)**, and the connections with the opposite direction (“task>rest”) are shown in blue in the right panel **(B)**.

Figure 3 shows the directed effective connectivity model of the DMN during the resting-state and the task-state learned by the BN approach. To better elucidate the organizational architecture of the DMN, we displayed the nodes including the PCC, MPFC, and bilateral IPC, which were called hubs in previous literatures (Buckner et al., 2008, 2009) in red, and other nodes in blue in the connectivity model graphs. In accordance with our previous BN analysis of the DMN (Li et al., 2012, 2013), Figure 3 demonstrates consistently in the two states that the non-hub regions including bilateral ITC and HC only receive connections from each other, and there is no connection generating from the PCC, IPC or MPFC to them. In the resting-state, the rITC receives connection from the lITC, and the lHC receives connections from the bilateral ITC and rHC; while in the task-state, the lITC receives connection from rITC, and the lHC receives connections from the bilateral ITC, and the rHC receives connections from the bilateral ITC and lHC. In contrast, the hub regions including the PCC, IPC, and MPFC not only receive connections from each other, but also receive connections from lITC and HC. That is the Bayesian network connectivity of DMN in both resting-state and task-state demonstrates a consistent “from non-hub subsystem to hub system” direction pattern. It is also important to note that the PCC works as a special node that does not generate but only receives connections in the network under both the two conditions. As shown in Figure 3, the PCC receives connections from the MPFC, rITC, rHC and bilateral IPC in both the two conditions and also receives two more connections from lHC and lITC during the resting-state.

### Between-group DMN effective connectivity difference

The results of the randomized permutation test are also shown in Figure 3, in which the connection weights in the resting-state group that are stronger than the ones in the task-state group (“rest>task”) are shown in blue in the Figure 3A, and the connections with the opposite direction (“task>rest”) are shown in blue in the Figure 3B.

Figure 3 demonstrated that the weight coefficients of the connections from lITC to rITC and bilateral IPC, from bilateral HC and MPFC to PCC, from lIPC to MPFC and PCC, from rIPC to lIPC, and from rHC to rIPC were greater during the resting-state as compared to the task-state condition. While during the task-state condition, the connections from bilateral ITC to MPFC and bilateral HC, from rITC to PCC and bilateral IPC, from MPFC to rITC and bilateral IPC, and from rIPC to PCC have larger connection weights than that during the resting-state condition.

## Discussion

In this study, we first detected the functional connectivity of the DMN in the resting-state and task-state conditions by using Group ICA. Then, we examined the functional connectivity difference between these two different conditions. The brain regions in the DMN that displayed significant reductions in the task-state group include the lIPC, rIPC, and PCC, whereas the lITC displayed significant reductions in the resting-state group. Furthermore, we employed a BN learning approach to explore the effective connectivity patterns of the DMN during the resting-state and the task-state. In conjunction with BN, we used a random permutation test to assess the effective connectivity group difference.

### Stable organizational architecture of the DMN

With or without a driving task, we found that the organization pattern of the DMN is basically stable in both the resting-state and task-state conditions, which is consistent with previous studies that report the stable organization pattern in the DMN (Friston, 1994; Smith et al., 2009; Ma et al., 2012; Li et al., 2013). In the present study, by referring to previous studies of the DMN (Buckner et al., 2008, 2009; Li et al., 2012, 2013), we divided regions in the network into hub regions including the PCC, MPFC, and IPC, and non-hub regions including the ITC and HC, and then examined the connections within the hub region subsystem, the non-hub region subsystem and the ones between them. The BN result of the DMN shows that, except for the interactions with each other, the ITC and HC do not receive any connections but only generate connections pointing to the PCC, MPFC and IPC during both conditions. While for the PCC, MPFC, and IPC, they are closely interconnected and also receive connections from other regions in the network. This directed connectivity relations suggest a consistent “from non-hub regions to hub regions” organization architecture which is in accordance with our previous BN analysis of the DMN in young (Li et al., 2012) and older (Li et al., 2013) subjects during the resting-state. The effective connections from the non-hub to hub regions suggest an orderly information transmission projecting from the lower areas to the higher areas during the resting and task state consistent with the recent models of cognition based on hierarchical Bayesian inference and Helmholtzian free-energy (Friston, 2003; Carhart-Harris and Friston, 2010). Here the result indicates that whether the brain is at rest or performing tasks, the DMN appears to remain a stable and similar organizational architecture. In addition we noticed that the PCC acted as a confluent node that integrated information from all other regions during both resting- and task-state conditions. It suggests that the PCC may play a pivotal role in mediating the neural activity throughout the whole network (Fransson and Marrelec, 2008). This is consistent with the observations of the PCC being a brain region of early and prominent amyloid pathology (Fransson and Marrelec, 2008) with reciprocal connections with the non-hub areas and connections with the prefrontal cortex and IPC (Kobayashi and Amaral, 2003, 2007).

The stability of the DMN in functional organizational architecture may first arise from the underlying solid neural anatomical infrastructure (Deco et al., 2010). Greicius et al. (2009) combined resting-state fMRI with diffusion tensor imaging (DTI) to demonstrate that functional connectivity of the DMN reflects the underlying structural connectivity. It is speculated that the neuroanatomical connections may develop an architecture to be able to store different and flexibly accessible brain functions (Deco and Jirsa, 2012). Thus, we could find that regions in the DMN were synchronously co-deactivated during a series of attention-demanding tasks while co-activated during the resting-state in previous reports (Raichle et al., 2001; Greicius et al., 2003; Raichle and Snyder, 2007; Buckner et al., 2008), and similarly we could demonstrate here that the dynamics of the DMN as represented by the BN directed connectivity maintained a stable architecture whether the brain was at rest or performing tasks. Smith et al. (2009) employed ICA to compare the functional connectivity networks during rest and activation, and they found a close correspondence between the independent analyses of activation networks and resting-state networks. Our study has further demonstrated a correspondence between the resting- and task-state dynamics and suggested a stable network organizational architecture that is based upon directed connectivity between regions in the DMN. The stabilized architecture is functionally meaningful in that it helps relevant DMN regions to process efficiently and respond fast to any external stimulations, and to be mobilized rapidly for perception and action (Deco et al., 2013). In addition, the result may also suggest that the DMN can be tested in a very short scanning session without having to decide in advance what experimental paradigm should be used and requiring active subject participation. This is particularly essential in the clinical settings where the subjects may be Alzheimer's patients.

### Differences of DMN effective connectivity between resting-state and task-state

Although the organization pattern of the DMN is basically stable, the interaction between different brain regions changed dramatically in the task-state compared with that in the resting-state. We found plenty of connections exert significant changes in these two states by comparing the difference of each connection weights. These findings indicate that the information processing mechanism of brain regions within the DMN was different between the resting-state and task-state, which may be related to the semantic task. Most worthy of mention is that among all the 12 connections that show increased connectivity weights during the task-state condition, nine of them are directly related to the ITG (Figure 3). This result is consistent with previous findings that the lateral temporal regions play an essential role in semantic processing (Demonet et al., 1992; Vandenberghe et al., 1996; Maguire and Frith, 2004; Wei et al., 2012). Many neuroimaging studies have demonstrated activations in the temporal regions in semantic-related visual (Kobayashi and Amaral, 2007) and auditory (Hickok and Poeppel, 2004) word and picture processing tasks. In stroke patients (Schwartz et al., 2009) and semantic dementia patients (Mummery et al., 2000), altered temporal structure was related to word-level semantic comprehensions. Wei et al. (2012) also found that the temporal regions functionally connected with the frontal cortex to generate a semantic network that largely overlapped with the DMN in configuration. The increased connectivity between the ITC and MPFC, PCC, bilateral IPC and HC as denoted by BN is highly consistent with the current recognition that the semantic processing is related with widely distributed regions including the medial and lateral temporal regions, prefrontal cortex, and posterior cingulate (Patterson et al., 2007; Binder et al., 2009; Han et al., 2013). Therefore, the increased directed connectivity suggests that the ITG was involved in the semantic processing together with the MPFC, PCC, IPC, and HC in the DMN.

### Limitations

Several limitations of the present study deserve a mention. First, as a DAG, BN cannot model reciprocal connections between different brain regions and self-influenced connections. The acyclic constraint on BN structure determines that the method cannot disclose reciprocal connections of the DMN in the present study. Second, the effective connectivity model constructed by BN is a single snapshot of the dynamic process, and it cannot explicitly disclose temporal causal relations between nodes. Future studies using the dynamic BN (Rajapakse and Zhou, 2007) which can capture temporal interrelationships of brain regions, and model reciprocal connections, would ideally be utilized to further compare the dynamic of the DMN during the resting-state and task-state conditions. Third, since the effective connectivity measures in the current study were estimated for the resting- and task-state groups separately, rather than individual subjects, it is difficult to establish correlations between fMRI connections and individual behavioral performance. Therefore, we note the connection directionality identified from BN should be cautiously interpreted.

In summary, using the BN learning approach, our current study explored the effective connectivity pattern of the DMN during the resting-state and the task-state. We have provided compelling evidence for the stable organization structure of the DMN whether the brain is in the resting-state or the task-state. In addition, we have also demonstrated that the interactions between different brain regions within the DMN are significantly changed in the task-state. The results suggest that the DMN intrinsically maintained a relatively stable structure whether at rest or performing tasks but had different information processing mechanisms under varied states. Furthermore, it is also our interest to explore the relationship of the connections between brain regions within the DMN and behavioral performance to further reveal the work mechanism of the DMN in the future.

### Conflict of interest statement

The authors declare that the research was conducted in the absence of any commercial or financial relationships that could be construed as a potential conflict of interest.
